# Unpredicted Pitch Modulates Beta Oscillatory Power during Rhythmic Entrainment to a Tone Sequence

**DOI:** 10.3389/fpsyg.2016.00327

**Published:** 2016-03-09

**Authors:** Andrew Chang, Dan J. Bosnyak, Laurel J. Trainor

**Affiliations:** ^1^Department of Psychology, Neuroscience and Behaviour, McMaster UniversityHamilton, ON, Canada; ^2^McMaster Institute for Music and the Mind, McMaster UniversityHamilton, ON, Canada; ^3^Rotman Research Institute, Baycrest HospitalToronto, ON, Canada

**Keywords:** sensory prediction, beta band, EEG oscillations, rhythmic entrainment, pitch, attention, auditory cortex, oddball

## Abstract

Extracting temporal regularities in external stimuli in order to predict upcoming events is an essential aspect of perception. Fluctuations in induced power of beta band (15–25 Hz) oscillations in auditory cortex are involved in predictive timing during rhythmic entrainment, but whether such fluctuations are affected by prediction in the spectral (frequency/pitch) domain remains unclear. We tested whether unpredicted (i.e., unexpected) pitches in a rhythmic tone sequence modulate beta band activity by recording EEG while participants passively listened to isochronous auditory oddball sequences with occasional unpredicted deviant pitches at two different presentation rates. The results showed that the power in low-beta (15–20 Hz) was larger around 200–300 ms following deviant tones compared to standard tones, and this effect was larger when the deviant tones were less predicted. Our results suggest that the induced beta power activities in auditory cortex are consistent with a role in sensory prediction of both “when” (timing) upcoming sounds will occur as well as the prediction precision error of “what” (spectral content in this case). We suggest, further, that both timing and content predictions may co-modulate beta oscillations via attention. These findings extend earlier work on neural oscillations by investigating the functional significance of beta oscillations for sensory prediction. The findings help elucidate the functional significance of beta oscillations in perception.

## Introduction

Perceptual systems extract regularities from the stream of continuous sensory input, and form internal representations for predicting future events. Predictive timing is the sensory prediction (or expectation) of when an event will occur (Nobre et al., 2007; Schroeder and Lakatos, 2009). Such predictions are hypothesized to be essential for many human behaviors, including understanding speech and music (Ding et al., 2015; Doelling and Poeppel, 2015), and synchronizing movements (Jenkinson and Brown, 2011; Fujioka et al., 2012, 2015; Kilavik et al., 2013). Predictive timing can be studied at a basic level in that an isochronous stream of metronome clicks sets up a strong prediction for when the next click will occur.

Entrainment is the process of internal neural oscillations becoming synchronized with temporal regularities in an external auditory rhythmic input stream, and it provides a mechanism for predicting future events in time (Jones, 2010). Such entrainment appears to be accomplished in the brain by neural oscillatory activity, which has been shown to represent temporal regularities in the sensory input, as well as the prediction of upcoming sensory events (Friston, 2005; Jones, 2010; Arnal and Giraud, 2012; Fujioka et al., 2012, 2015; Henry and Herrmann, 2014; Morillon and Schroeder, 2015; Herrmann et al., 2016). While time domain event-related potential (ERP) analyses of electroencephalogram (EEG) waveforms in response to unpredicted stimuli have revealed aspects of neural processes underlying sensory prediction (e.g., Costa-Faidella et al., 2011; Schwartze and Kotz, 2013; Schröger et al., 2015), recent studies indicate that neural oscillatory activities obtained by decomposing EEG signals into frequency-specific bands reveal processes of communication between neural ensembles (Buzsaki, 2006) that are essential to sensory prediction (Arnal and Giraud, 2012).

Oscillatory activities in sensory cortices in both delta (1–3 Hz) and beta (15–25 Hz) bands are associated with temporal prediction (Henry and Herrmann, 2014). The phase of the delta oscillation shows entrainment to rhythmic sequences and it is reset by the onset of a stimulus and predicted (imagined) onset of a future stimulus. On this basis, it has been suggested that delta phase reflects an oscillatory time frame for parsing a continuous sensory stream into meaningful chunks for subsequent perceptual processing (Schroeder and Lakatos, 2009; Calderone et al., 2014). Neural responses to sensory inputs that occur at the time of the excitation phase of delta oscillations are enhanced compared to those that coincide with the inhibition phase (Schroeder and Lakatos, 2009). Local field potential recordings in primary visual and auditory cortices of macaque monkeys show that the delta phase entrains to the onsets of stimuli in rhythmic stimulus streams (Lakatos et al., 2008, 2013), consistent with intracranial electrocortical and surface EEG recordings in humans (Besle et al., 2011; Gomez-Ramirez et al., 2011; Henry and Obleser, 2012; Herrmann et al., 2016), and it can be endogenously directed by selectively attending to one or the other of two simultaneous stimulus streams (Lakatos et al., 2008, 2013; Calderone et al., 2014).

**FIGURE 1:**
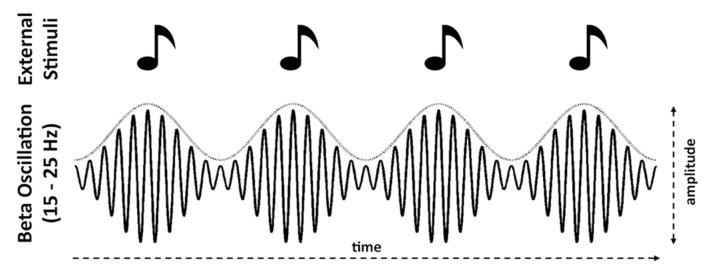
**Schematic illustrations of power modulation in induced (non-phase-locked) beta (15–25 Hz) entraining to the tempo of the stimuli.** Specifically, power decreases following isochronous onsets and increases that predict the onset time of the next stimulus (e.g., Fujioka et al., 2012; Cirelli et al., 2014). The dotted curve above the beta waveform envelope represents this power modulation.

The amplitude fluctuation dynamics of induced (non-phase-locked) beta band power also entrain to the tempo of events in an auditory input stream, as well as reflecting temporal prediction. EEG and MEG recordings of isochronous auditory sequences show that induced beta power decreases following each tone onset, and increases again prior to the onset time of the next tone, with the timing of the increase varying with tempo in a predictive manner (Snyder and Large, 2005; Fujioka et al., 2009, 2012, 2015; Iversen et al., 2009; Cirelli et al., 2014; **Figure 1**). Both delta phase angle and beta power in auditory and motor areas in the pre-stimulus onset period predict the accuracy of detecting a temporal delay in the stimulus (Arnal et al., 2015). Furthermore, in primary motor cortex, beta power is modulated by attention, and aligned with the delta phase, suggesting that beta power might reflect attentional fluctuation in time and delta phase an entrained internal clock that aids in the execution of a motor task (Saleh et al., 2010).

Although delta phase and induced beta power are both associated with temporal prediction, compared to the compelling evidence for delta oscillations, the functional significance of beta oscillations in perceptual processing remains less clear. We hypothesized that the entrainment of induced beta power in auditory cortex to an external stimulus might reflect more than predictive timing. Specifically, given that auditory cortex is sensitive to both spectral and temporal dimensions of the input (Fritz et al., 2003; Griffiths and Warren, 2004; King and Nelken, 2009), and auditory evoked ERP components can be interactively modulated by predictions of both pitch and time (Costa-Faidella et al., 2011), beta oscillations might also reflect predictive coding for specific content, such as pitch. In order to examine this hypothesis, we conducted two experiments in which we presented isochronous auditory oddball sequences containing occasional deviations in pitch at different presentation rates. If the induced beta power only reflects predictive timing, the occasional unpredicted pitch changes should not affect the ongoing beta entrainment behavior, given that the pitch deviants are presented at the predicted rhythmic time points. On the other hand, if the induced beta power is affected by the unpredicted deviant pitches, it would suggest that beta power is associated with predictive perceptual processing for both what and when. In the case that induced beta power is affected by unpredicted deviant pitches, we examine further whether it is modulated by response to novelty (rare events in the preceding local context) or prediction error (the probability of encountering a deviant pitch under the statistical conditions of the context).

## Materials and Methods

### Stimuli

Two recorded piano tones, C4 (262 Hz) and B4 (494 Hz), from the University of Iowa Musical Instrument Samples were used. The amplitude envelopes of the piano tones were percussive with 10 ms rise times. Tones were truncated to be 200 ms in duration, and a linear decay to zero was applied over the entire excerpt to remove offset artifact. The DC shift was removed for each tone. Sounds were converted into a monaural stream at 71 dB (C weighted), measured through an artificial ear (type 4152, Brüel & Kjær) with sound level meter (type 2270, Brüel & Kjær).

### Procedure

The experiment was conducted in a sound-attenuated room. Each participant was presented with a continuous sequence of tones in two sessions, each lasting 30 min, while they watched a silent movie on a computer screen. Participants took a 3-min break between sessions. Sounds were delivered binaurally via ear inserts (Etymotic Research ER-2). All stimulus sequences were presented under the control of a digital signal processor (Tucker Davis RP2.1).

The tones were presented in an oddball sequence. The C4 tone was used as the standard and the B4 tone as the deviant. For the first group of participants, the inter-onset interval (IOI) was fixed at 500 ms. There were 3600 tones presented in each session, and the deviance occurrence rate was 10% in one session and 20% in the other session, with an equal number of participants completing the 10% or 20% session first. Within each session, tone order was pseudorandomized with the constraint that two deviant tones could not be presented sequentially, and each session started with five consecutive standard tones. Participants were instructed to sit comfortably and remain as still as possible during the experiment while watching a silent movie. They were not required to make any responses.

In order to replicate and to generalize the findings to a different presentation rate, for a second group of participants, we employed a longer IOI of 610 ms in an isochronous oddball sequence with the 10% deviant tones condition. Otherwise, the procedure for group two was the same as that for group one.

For convenience, we refer to the 500 ms IOI experimental sessions (10% and 20% deviance occurrence rates) as the Fast Experiment, and the 610 ms IOI experimental session (10% deviance occurrence rate only) as the Slow Experiment.

### Participants

Sixteen participants (17–22 years old, mean age 18.93 ± 1.39; 12 female) for the Fast Experiment and a different thirteen participants (17–21 years old, mean age 18.62 ± 1.33, 10 female) for the Slow Experiment were recruited from the McMaster University community. Participants were screened by a self-report survey to ensure they had normal hearing, were neurologically healthy and were right-handed. Signed informed consent was obtained from each participant. The McMaster University Research Ethics Board approved all procedures. Participants received course credit or reimbursement for completing the study.

### Electroencephalographic Recording

The EEG was sampled at 2048 Hz (filtered DC to 417 Hz) using a 128-channel Biosemi Active Two amplifier (Biosemi B.V., Amsterdam). The electrode array was digitized for each participant (Polhemus Fastrak) prior to recording. EEG data were stored as continuous data files referenced to the vertex electrode.

### Signal Processing of the EEG Data

Three stages of signal processing were conducted in order to examine the behavior of auditory evoked and induced oscillations in bilateral auditory cortices. In the first stage, we obtained a dipole source model based on auditory evoked responses, following Fujioka et al. (2012). The second stage segmented and categorized the source waveform into epochs based on the relative order of the presented auditory sequence. In the third stage, epochs containing excessive artifacts were rejected.

#### Stage 1: Dipole Source Modeling

**FIGURE 2:**
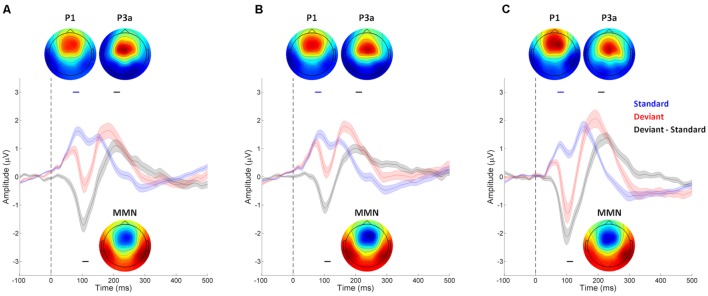
**Auditory evoked event-related potential (ERP) waveforms of mid-frontal electrodes from the **(A)** 10% session and **(B)** 20% session of the Fast Experiment, and **(C)** Slow Experiment.** Waveforms were collected using 128 EEG channels, and averaged across channels located at the mid-frontal area (F1, Fz, F2, FC1, FCz, and FC2), with stimulus-onset at 0 ms (indicated by the vertical dashed line in each plot). The shaded areas indicate the SEMs of standard trial (blue), deviant trial (red), and the difference waveform of deviant minus standard trial (black). The ERP waveforms of standard trials show a prominent P1 component around 70–90 ms (indicated by the blue line above each waveform). P1 topography of each session (inset; red represents positive potential, blue negative) shows a mid-frontal focus, consistent with generators in primary auditory cortex. The ERP difference waveforms show significant MMN (100–120 ms, indicated by the black line below each waveform) and P3a (200–220 ms, indicated by the black line above each waveform) components. The topography of the MMN (inset) shows the typical frontal negativity of the MMN. The P3a is larger in deviant than standard trials (inset), with typical topography showing a frontal positivity.

The continuous EEG data was band-pass filtered 0.3–100 Hz for each participant for each session, and then segmented into epochs covering the time period -100 to 300 ms, time locked to stimulus onset. Epochs containing standard tones that preceded and followed other standard tones with amplitudes exceeding 150 μV were rejected as artifacts. The surviving standard epochs (89.6% ± 5.1% for 10% session and 89.5% ± 5.1% for 20% session of Fast Experiment, and 88.4% ± 5.5% of Slow Experiment) were averaged into ERP waveforms and band pass filtered between 1 and 20 Hz (**Figure 2**). To confirm that our oddball context was set up appropriately, a similar procedure was performed on the deviant epochs, and the average of the standard epochs subtracted from the average of the deviant epochs in order to produce difference waves. As can be seen in **Figure 2**, both mismatch negativity (MMN) and P3a responses can be observed, consistent with the literature on ERP responses in oddball contexts (Friedman et al., 2001). Paired *t*-tests, performed on the average of channels in the mid-frontal area (F1, Fz, F2, FC1, FCz, and FC2), confirmed the presence of an MMN component between 100 and 120 ms; specifically, deviant trials were significantly more negative than standard trials in this time window in all sessions of both Fast and Slow Experiments (*p*s < 0.001). There was also a P3a component between 200 and 220 ms: deviant trials were significantly more positive than standard trials in this time window in all sessions of both Fast and Slow Experiments (*p*s < 0.001). It is worth noting that although the latencies of MMN and P3a observed in the current study were earlier than are sometimes reported (e.g., MMN: 150–250 ms, P3a: 250–300 ms; Friedman et al., 2001; Näätänen et al., 2007; Polich, 2007), our results are consistent with several previous studies showing that the latencies of MMN and P3a are as short as around 100 and 200 ms, respectively, when the stimuli are presented in a rhythmic context with IOIs less than or equal to 700 ms (e.g., Regnault et al., 2001; Jongsma et al., 2004; Pablos Martin et al., 2007; Matsuda et al., 2013).

**FIGURE 3:**
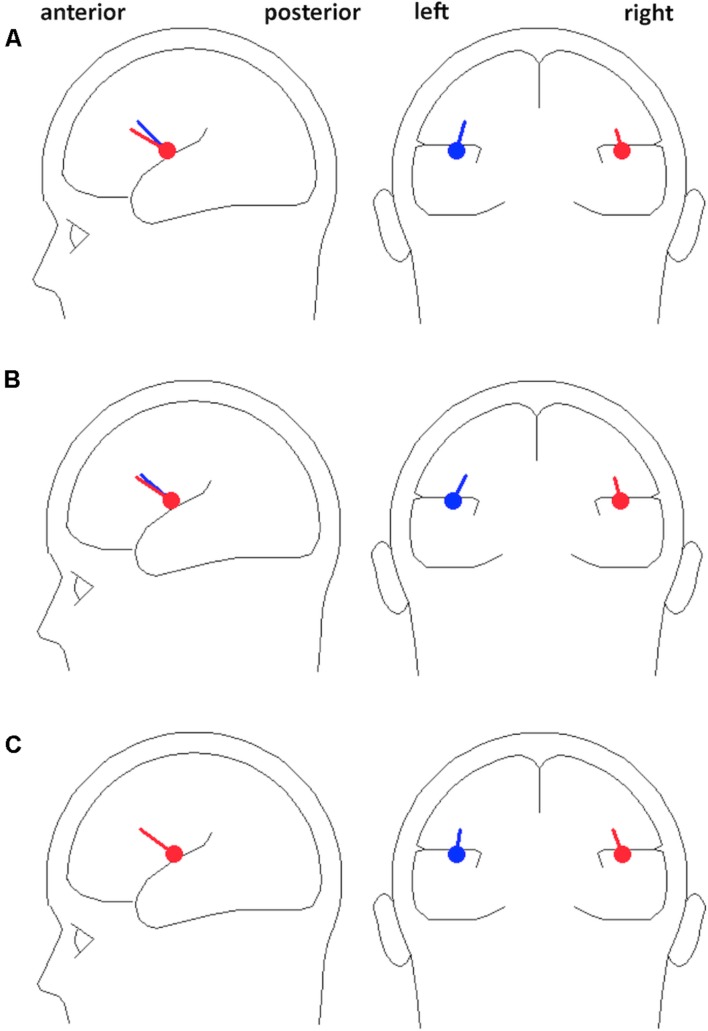
**The mean locations and orientations of dipoles.** Dipole locations were symmetrically fitted for the auditory P1 ERP component across participants for each **(A)** 10% session and **(B)** 20% session of the Fast Experiment, and **(C)** Slow Experiment, presented in both sagittal and coronal planes. The fitted dipoles are closely located at bilateral primary auditory cortices with orientations toward frontal midline.

We employed a dipole source model as a spatial filter for increasing the signal-to-noise ratio of the EEG signal generated from left and right auditory cortices for subsequent analyses. A previous study showed that beta activities generated in both auditory and motor cortices entrained to external auditory rhythms when participants passively listened to isochronous sequence of tones (Fujioka et al., 2012). In the present study, we were primarily interested in responses from auditory areas, so we analyzed the EEG signals in source space rather than from surface channels, to extract the oscillatory signals generated from auditory cortex while attenuating signals generated from other brain regions. The source modeling was performed on each participant’s mean standard ERP waveform using the multiple source probe scan algorithm and the four-shell ellipsoid model included in the Brain Electrical Source Analysis (BESA) software package. Two auditory cortex sources were estimated for each participant for the auditory evoked P1 (60–100 ms; **Figure 2**) with the dipoles constrained to be symmetric across hemispheres in location but not orientation. P1 was chosen because it is the dominant peak at fast presentation rates (N1 peaks are strongly reduced at fast rates; Näätänen and Picton, 1987), and is generated primarily from primary auditory cortex (Godey et al., 2001). The mean locations of fitted dipoles across participants were at Talairach coordinates -45.0, -3.2, 16.2 with orientation (0.2, 0.6, 0.8) and 45.0, -3.2, 16.2 with orientation (-0.1, 0.7, 0.7) in the 10% session of the Fast Experiment; and at -45.4, -3.1, 17.2 with orientation (0.3, 0.7, 0.7) and 45.4, -3.1, 17.2 with orientation (-0.1, 0.8, 0.6) in the 20% session of the Fast Experiment; and -44.9, -4.7, 16.4 with orientation (0.1, 0.7, 0.7) and 44.9, -4.7, 16.4 with orientation (-0.2, 0.7, 0.7) in the Slow Experiment, which are all closely located at bilateral primary auditory cortices with orientations toward the mid-frontal surface area (**Figure 3**). The residual variances of the source fittings for each session for each participant were between 5% and 10%.

#### Stage 2: Epoching

Based on individual participant dipole model fits for each session, the source activities of single trials in auditory cortices were extracted for all epoch types using signal space projection following Fujioka et al. (2012). Because we were interested in the inter-stimulus neural responses, and to avoid edge effects in subsequent time-frequency analysis, the unfiltered EEG data of each session were segmented into relatively long -500 to 1000 ms epochs, where 0 ms represents a stimulus onset. The epochs were categorized based on the relative position of tones presented in the experiment, including standard (standard tones between two standard tones), deviant (deviant tones between two standard tones) and SpreD (standard tones preceding a deviant tone and following a standard tone). The individual source waveform epochs as well as raw channel EEG data were exported from BESA to MATLAB for further processing.

#### Stage 3: Artifact Rejection

Another artifact rejection procedure was applied to the raw 128-channel data. Epochs identified to have artifacts were noted, and the corresponding source waveform epochs were eliminated from further analysis. Thus we made sure the source waveform epochs entered into the time-frequency analysis in the next stage were artifact-reduced and unfiltered, to maximize the signal-to-noise ratio. Because we aimed to reject epochs containing EOG or EMG responses, each raw channel EEG epoch was filtered by a third-order Butterworth band pass filter (1–60 Hz). The filtered EEG epochs that exceeded a threshold (40 μV, compared to the baseline mean voltage of -100–0 ms) for more than 10% of the epoch at any channel were excluded from further analysis. An additional seven participants’ data were not included in the current data set because more than 50% of their epochs did not pass the criteria at this stage. For the remaining participants 66.18% ± 8.68% of the epochs in the Fast Experiment and 71.57% ± 10.54% in the Slow Experiment were accepted for further analysis.

### Time-Frequency Decompositions

Time-frequency decompositions were calculated for each participant on each single-epoch source waveform in left and right auditory cortices and for each stimulus condition using a Morlet wavelet transform (Bertrand et al., 1994) for beta frequency band.

In order to remove the evoked (phase-locked) responses from the epoch and thereby obtain the induced (non-phase-locked) responses for subsequent analyses on beta band, we averaged the source waveform for each trial type (evoked response estimate), and then subtracted it from each source waveform epoch (Kalcher and Pfurtscheller, 1995; Fujioka et al., 2012).

The Morlet wavelet transformation was calculated for each time point for each induced epoch with 32 logarithmically spaced frequency bins between 15 and 25 Hz. The wavelet was designed such that the half-maximum width was equal to 3.25 periods of the lowest frequency while the width was equal to 3.56 periods of the highest frequency, linearly interpolated for each frequency bin in between. Subsequently, 300 ms at the beginning and ending of the epoch were eliminated to avoid edge effects. The induced oscillatory mean signal power was calculated by averaging the magnitude of each time-frequency point of wavelet coefficients across trials. Normalizing this to the mean value of the standard epochs across the whole epoch for each frequency resulted in relative signal power changes expressed as a percentage (Fujioka et al., 2012), and all types of epochs within the same session were compared to the same baseline (mean power in the averaged standard epoch between 0 and 500 ms). The fluctuation in power for each type of epoch at each frequency was visualized as a function of time and frequency in color-coded maps of event-related synchronization and desynchronization (Pfurtscheller and Lopes da Silva, 1999).

### Discrete Fourier Transform for Neural Oscillation Entrainment

In order to examine whether the observed neural oscillation activity entrained to the presented stimulus rate, we analyzed the time series of each participant’s normalized mean induced beta power (derived as above) via discrete Fourier transforms (DFT). For each participant, we took the -200 to 700 ms epoch for the averaged induced beta power from the wavelet transform, zero-padded to 5 s in order to increase the frequency resolution of the DFT to a bin size 0.2 Hz. For each of the beta power time series, the power spectrums revealed by the DFTs were averaged across participants at each of the left and right auditory cortices.

### Data Analysis and Statistics

In order to examine whether the deviant tone affected the beta band induced power (1) we compared the standard and deviant trials for each individual participant for both the 10% and 20% deviance sessions to identify deviant-elicited prediction error responses, and (2) we compared this difference of “standard - deviant” between the 10% and 20% deviance rate sessions to investigate the effect of prediction precision, as deviants in the 10% session are less predicted than those in the 20%. We analyzed the window 0–500 ms for the Fast Experiment and 0–610 ms for the Slow Experiment, time-locked to stimulus onset. The standard and deviant trials of individual participants were then used for random effects analysis.

To assess the statistical differences between the induced beta band powers while controlling for multiple comparisons, we performed cluster-based permutation analyses on the two-dimensional time-frequency maps (Maris and Oostenveld, 2007). First, we used a Wilcoxon signed-rank test, a non-parametric paired difference test, to examine the mean power difference in the beta band between each paired time-frequency sample from 0 to 500 ms for the Fast Experiment or 0–610 ms for the Slow Experiment. Second, we grouped the time-frequency adjacent samples reaching a threshold of *p* < 0.05 into single clusters. Third, we summed the test statistics within each cluster into a cluster-level statistic, which became the observed value. Fourth, to build a permutation distribution, we randomly interchanged the experimental conditions for each participant, repeated the previous three steps 5000 times, and extracted the largest cluster-level statistics for each repetition. The final p-value was calculated by comparing the observed value of each cluster with the permutation distribution.

## Results

**FIGURE 4:**
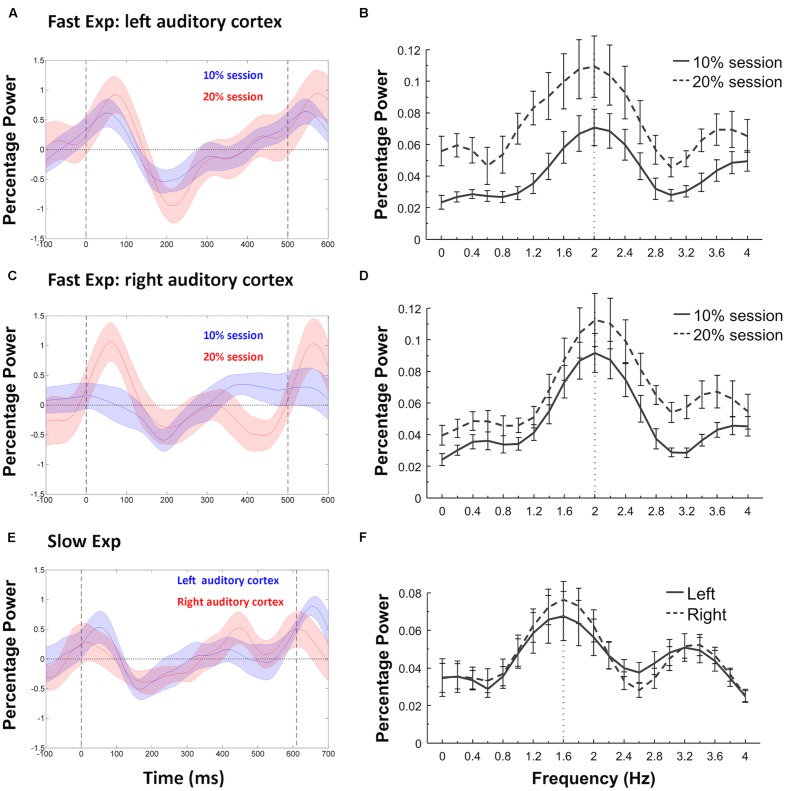
**Power fluctuations of induced beta (15–25 Hz) and associated discrete Fourier transformation (DFT) analyses.** Fast Experiment: **(A)** shows the induced beta power fluctuations in the standard trials of the 10% and 20% sessions in left auditory cortex, with shaded areas indicating SEM and vertical dashed lines representing the onsets of tones at 0 and 500 ms. The induced beta power decreases after the onset of a standard tone, and increases (or “rebounds”) again before the onset of the next tone. The DFT analyses **(B)** confirmed entrainment to the stimulus presentation rate (dashed lines) in each case, with maximum power at 2.0 Hz. The same results were replicated at the right auditory cortex **(C,D)** of the Fast Experiment. Slow Experiment: **(E)** shows the induced beta power fluctuations in both left and right auditory cortex, with the vertical dashed lines representing the onsets of tones at 0 and 610 ms. The DFT analyses **(F)** confirmed that the power entrained to the stimulus presentation rate (dotted lines), with maximum power at 1.6 Hz.

We first tested whether the induced beta power entrainment phenomenon reported by Fujioka et al. (2012) was replicated in the standard trials. In the Fast Experiment, the induced power in the beta band of the standard trials showed a clear entrainment to the IOI rate (2.0 Hz). Specifically, the DFT analysis on induced beta band power showed the strongest power at 2.0 Hz for both the 10% and 20% sessions at both left and right auditory cortices (**Figures 4A–D**). In the Slow Experiment, the induced power in the beta band of the standard trials showed a clear entrainment to the slower IOI rate (~1.6 Hz) with the DFT analysis showing the strongest power at 1.6 Hz at both left and right auditory cortices (**Figures 4E,F**). These results replicate previous studies showing that induced beta band power entrains to the IOI of isochronous stimulus sequences (Fujioka et al., 2009, 2012, 2015; Cirelli et al., 2014).

**FIGURE 5:**
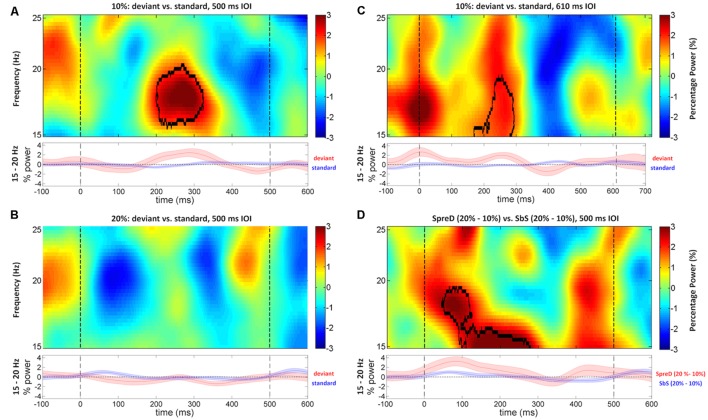
**Time-frequency maps of induced power difference between deviant and standard trials in the beta frequency range (15–25 Hz) at right auditory cortex of Fast and Slow Experiments.** The shaded areas under each time-frequency map indicates SEM of the low-beta (15–20 Hz) power fluctuations. In the Fast Experiment, standard/deviant tones begin at 0 ms, and the onset time of the next tone is 500 ms (dashed lines). The black contours represent the significant time-frequency cluster. **(A)** The difference between time-frequency maps (deviant minus standard trials) shows that the deviant tone in the 10% sessions induced stronger power compared to the standard at right auditory cortex, around 16–20 Hz and 200–300 ms. **(B)** The difference between time-frequency maps (deviant minus standard trials) did not show any significant difference in the 20% session at right auditory cortex. **(C)** In the Slow Experiment, the standard/deviant tones begin at 0 ms, and the onset time of the next tone is 610 ms (dashed lines). The difference between time-frequency maps (deviant minus standard trials) shows that the deviant induced stronger low-beta (15–19 Hz) power compared to the standard at right auditory cortex, around 200–300 ms. **(D)** This shows the subtraction of the two difference maps SpreD trials (20% minus 10%) minus SbS trials (20% minus 10%) of Fast Experiment. The result showed that the power difference is larger between SpreD trials than between SbS trials across sessions, around 15–19 Hz and 50–250 ms.

We then examined whether trial type (deviant vs. standard) and session (deviant rate) modulate the induced beta power, in additional to the entrainment activities. In the Fast Experiment, the cluster-based permutation test identified one significant cluster in the 10% session at right auditory cortex, in which the mean induced power at 16–20 Hz, within the range of low-beta band (15–20 Hz), around 200–300 ms after stimulus onset was larger in the deviant trials than the standard trials (*p* = 0.044; **Figure 5A**) with a large effect size (rank correlation = 0.67). We did not identify any significant cluster at left auditory cortex. We examined the same contrast for the 20% session. Although we failed to identify any significant cluster at either left or right auditory cortex, the power difference of “deviant–standard” trials peaked around 200–300 ms in the low-beta band at right auditory cortex (**Figure 5B**), which is consistent with the results of the 10% session. We further compared the power difference of “deviant–standard” trials between the 10% and 20% sessions at the previously identified cluster. The Wilcoxon signed-rank test showed that the power difference was significantly larger in the 10% session than in the 20% session (*p* = 0.026), with a large effect size (rank correlation = 0.56). Taken together, this indicates that the induced power in low-beta band around 200–300 ms after stimulus onset was higher in deviant trials than in standard trials, and that this effect was larger in the 10% session than in the 20% session.

The results of the Slow Experiment replicated the results of the Fast Experiment. A cluster-based permutation test showed only one significant cluster around 200–300 ms after stimulus onset at 15–19 Hz at right auditory cortex (*p* = 0.026; **Figure 5C**), in which the mean induced power was larger in the deviant trials than the standard trials with a large effect size (rank correlation = 0.79).

To further distinguish whether the deviant-induced responses in low-beta band are associated with prediction error or response to novelty (rare events in the preceding local context), given that both processes can be engaged by deviant stimuli in an oddball context (Friedman et al., 2001), we performed an additional analysis for standard tones occurring in different places in the sequence. This was based on the idea that in an oddball sequence, not only can the presentation of a deviant tone violate a prediction for a standard tone, but also the presentation of a standard tone that follows several standard tones in a row can violate an expectation (prediction) for a deviant tone. Specifically, the more standards that occur in a row, the more likely it is that a deviant will occur next, given a fixed overall probability of a deviant. On the other hand, a standard occurring after several standards in a row would not elicit a novelty response, as there is no change in the stimulus. If the beta band response that we measured reflects prediction error and not response to novelty, then the response to standard tones should depend on how many standards occurred prior to the standard of interest (as each successive standard builds prediction for an eventual deviant), whereas if the response simply associates with novelty, there should be a larger response to standards in the 20% than 10% condition, but no effect of how many standards occur in a row. Given that a deviant tone must occur eventually along the time line (Luce, 1986; Nobre et al., 2007), the conditional likelihood of encountering a standard tone decreases with the number of repetitions of the standard tone in a row, and thus, on average, the prediction of standard tones preceding a deviant tone will be lower in the 10% than in the 20% session since there are on average more standards in a row before each deviant in the 10% condition.

We can compare responses to standards between 10% and 20% sessions that occur either immediately before a deviant (SpreD) or between two other standards in the sequence (here referred to as SbS). SbS trials occur earlier on average in the sequence compared to SpreD trials. This allows a test of the two alternative hypotheses. Specifically, if the induced low-beta power response at right auditory cortex results from prediction error, the power difference between SpreD trials (20% session–10% session) should be larger than the difference between SbS trials (20% session–10% session), because the prediction error (mismatch between standard and deviant tone) is modulated by conditional likelihood (the position of standard tones in a stimulus sequence). On the other hand, if the induced low-beta power response is modulated by the novelty in the preceding context, the power difference between SpreD trials (20% session–10% session) should be equal to the difference between SbS trials (20% session–10% session), because the conditional likelihood does not matter. Indeed, if anything, the SbS trials would be predicted to show a larger induced low-beta power difference than the SpreD trials because the SbS trials constitute a deviation from a more recently presented deviant tone whereas SpreD trials follow a larger number of standard trials. A cluster-based permutation test in low-beta band at right auditory cortex showed that the SpreD trials had a larger induced power difference than the SbS trials (*p* = 0.045; **Figure 5D**) around 50–250 ms at 15–19 Hz with a large effect size (rank correlation = 0.74). This suggests that the increased induced low-beta power is elicited by prediction error, modulated by conditional likelihood, rather than response to novelty, modulated by rareness of a pitch in the preceding context.

**FIGURE 6:**
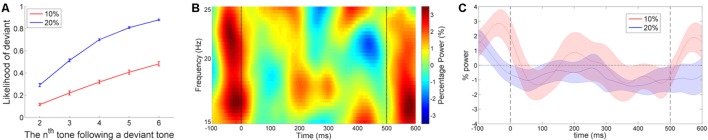
**The cumulative conditional likelihoods of encountering a deviant tone, and the time-frequency maps of induced difference (deviant minus standard) responses on matched trial locations in the beta frequency range (15–25 Hz) at right auditory cortex between the 10% and 20% sessions of Fast Experiment. (A)** The cumulative conditional likelihoods of encountering a deviant tone as a function of the n^th^ location following a deviant trial in 10% session (red) and 20% session (blue) with error bar indicating SEM. This was calculated by summing up the empirical occurrence rates of deviant tones at the current location and all preceding locations in the experiment. The likelihood of a deviant tone being presented at the n^th^ location is the accumulation of the occurrence rate from the first to n^th^ location following the previous deviant trial. **(B)** The subtraction of the two difference maps in the 10% session (deviant minus standard) minus the 20% session (deviant minus standard) at the second to the sixth trial following a deviant tone. Although the cluster-based permutation test did not find any cluster to be significantly different, the maximum of low-beta power difference (deviant minus standard) within the 130 to 370 ms window, time-locked to stimulus onset, was significantly larger in the 10% session than in the 20% session. **(C)** The shaded areas indicate SEM of the averaged low-beta (15–20 Hz) power difference (deviant minus standard) fluctuations of 10% session (red) and 20% session (blue).

Another additional analysis was performed to investigate whether the current results were associated with the mechanism of auditory stimulus-specific adaptation (SSA) rather than sensory prediction. Auditory SSA refers to the phenomenon that the neural response to the same tone decreases as the number of times it is repeated increases, and raises the possibility that responses to rare tones in an oddball context reflect release from adaptation rather that prediction or response to novelty (e.g., Butler, 1968; Näätänen et al., 1988; Lanting et al., 2013). In the present study, it is possible that the magnitude of the low-beta response to pitch deviants reflects a release from adaptation to the repeated standard tones in our oddball context. Further, the finding that the low-beta power response was stronger on deviant trials in the 10% than 20% session might be due to the fact that there were on average more repeated standard tones preceding a deviant trial in the former case. In order to investigate whether the low-beta response was modulated by a predictive process, we compared conditions where the effect of SSA was constant, but prediction differed. Specifically, to accomplish this, we compared 10% and 20% sessions of the Fast Experiment where the number of standards since the previous deviant was held constant. Thus, we averaged separately deviant effects where there were two standards, three standards, four standards, five standards, or six standards since the last deviant. In each case, we took the low-beta power difference of deviant minus standard trials and compared between the 10% and 20% sessions. The critical point is that, for a given number of standard trials preceding a deviant, the sensory prediction hypothesis indicates that deviants are more expected in the 20% than 10% session because there is a generally higher probability of a deviant in the 20% condition. Specifically, the conditional likelihoods of encountering a deviant tone can be estimated by summing up the empirical occurrence rates of a deviant tone in the all the locations in a sequence following a deviant trial, until the current location (**Figure 6A**). We performed a cluster-based permutation test on the low-beta band at right auditory cortex. We did not find any cluster to be significant, but there was a trend for the power difference at the cluster at 200 to 300 ms to be larger in the 10% session than in the 20% session (**Figure 6B**) as predicted by the sensory prediction hypothesis. The fact that it did not reach conventional significance levels is likely due to the small number of trials (in the 10% session, 141.0 ± 19.0 deviant trials were included in the current analysis, compared to the 244.6 ± 37.8 trials that were included in previous analyses). We compared the maximum deviant minus standard power difference of the averaged low-beta frequency band between 10% and 20% sessions in the time window 130–370 ms for each participant, time-locked to stimulus onset (**Figure 6C**). The Wilcoxon signed-rank test showed that the maximum low-beta power difference between deviant and standard trials was significantly larger in the 10% session than in the 20% session (2.96 ± 1.09 vs. 0.32 ± 0.45, *p* = 0.040) with a medium effect size (rank correlation = 0.53). This suggests that the increased induced low-beta power is associated with the degree of prediction error when we controlled the effect of SSA to be the same in both sessions.

In sum, we showed that the deviant tone induced an increase in power in the low-beta band around 200–300 ms following tone onset in right auditory cortex, regardless of the presentation rate. Also, the effect was stronger when the deviance occurrence rate was lower. Furthermore, two additional analyses suggest that the induced low-beta power was higher for standard tones that violated a stronger prediction for a deviant tone, confirming that the low-beta response is more likely to reflect prediction error than response to novelty. Also, the induced low-beta power response was larger on deviant trials when they were less predictable, even when the effects of SSA were controlled, again suggesting that the low-beta response to deviant tones reflected processes associate with prediction.

## Discussion

We sought to understand the roles of beta oscillations in entrainment to rhythmically predictable sequences by introducing occasional unpredictable pitch deviants. We replicated previous findings related to timing entrainment in induced beta power (Snyder and Large, 2005; Fujioka et al., 2009, 2012, 2015; Iversen et al., 2009; Cirelli et al., 2014), showing that fluctuations in beta power entrained to the rate of presented isochronous auditory stimulus sequences in both left and right auditory cortices. In addition, we found that induced beta band power at right auditory cortex increased around 200–300 ms after the onsets of deviant tones compared to standard tones, especially in the low-beta range (15–20 Hz). This effect was larger when the deviant pitch was less likely to occur (10% vs. 20%), suggesting it is related to prediction processes. The right lateralization of the beta response to pitch deviants is consistent with the idea that the right auditory cortex is more sensitive for processing spectral information than its left counterpart (e.g., Zatorre et al., 1992, 2002). To the best of our knowledge, this is the first study to show that induced beta power in auditory cortex is sensitive to an unpredicted pitch change, even when it is presented at the predicted time. This suggests that induced beta power plays a role in sensory prediction for both *what* will occur as well as *when* it will occur.

The increased beta response with decreased likelihood of deviance occurrence indicates that beta oscillations may associate with precision-weighted prediction error. It has been suggested that while prediction error signals do not necessarily involve attention, high precision-weighted prediction errors act through attention to increase the gain of neural responses, acting as teaching signals for subsequent prediction updating (Friston, 2009; den Ouden et al., 2012; Hohwy, 2012; Schröger et al., 2015). According to predictive coding theory, prediction error is defined as the sensory mismatch between the predicted and perceived stimuli, and precision is the inverse of the input variance of the context which determines whether or not to deploy attention for updating future predictions (den Ouden et al., 2012). For example, prediction precision is higher for standard tones in the 10% than 20% session, because on average there are fewer deviant tone are intermixed in the same length of sequence in 10% than 20% sessions. Thus, larger beta power responses to deviants in the 10% compared to 20% session might indicate that the process involved is one of prediction precision. That beta oscillations are associated with deploying attention for improving perceptual performance is supported by attentional blink studies showing that enhanced phase synchronization in low-beta band among frontal–parietal–temporal regions involved in the attentional network is associated with improved behavioral performance for targets with abrupt onsets (Gross et al., 2004; Kranczioch et al., 2007). Further, it has also been suggested that gamma oscillations (>30 Hz) reflect feed forward prediction error signals (Herrmann et al., 2004) while beta oscillations represent a subsequent feed back processing stage for updating prediction (Arnal and Giraud, 2012), again consistent with the idea presented here that low-beta is sensitive to the precision of prediction, and associates with attention and prediction updating.

The latency of the low-beta response also implies that it is likely associated with attention and prediction updating. The low-beta response to pitch deviants in our data was around 200–300 ms after tone onset, which was later than the well-studied MMN prediction error response in the time waveform ERP, which was around 100 to 120 ms (**Figure 2**), consistent with other studies employing rhythmic sequences with relatively fast IOIs (Näätänen et al., 2007; Pablos Martin et al., 2007; Fujioka et al., 2008; Matsuda et al., 2013; Hove et al., 2014). This suggests that the low-beta response reflects a processing stage that is later than detecting prediction error. Interestingly, the 200–300 ms timing of the beta band power response occurs around the same time as P3a (Regnault et al., 2001; Jongsma et al., 2004, see **Figure 2** for P3a latency), which is known to reflect exogenous attentional orienting and attentional updating (Friedman et al., 2001; Polich, 2007). The P3a and induced low-beta power likely reflect distinct neural responses because the P3a is phase-locked to stimulus onset and originates in the anterior cingulate cortex and related structures (Polich, 2007) while, in contrast, the induced low-beta power response is not phase locked to stimulus onset and is observed with a spatial filter located in auditory regions. However, the overlapped response latencies are consistent with the idea that attentional processing in frontal areas, reflected by P3a, interacts with prediction precision, and is associated with induced beta power in auditory cortex.

To further evaluate the idea that beta is associated with precision-weighted prediction error, it is important to consider the alternative possibility that the beta band power increases we observed following pitch deviants are simply a response to novelty in the preceding local context rather than prediction error. Indeed, a number of studies in humans and other animals have shown effects of rare stimuli on both induced and evoked beta oscillations (Haenschel et al., 2000; Kisley and Cornwell, 2006; Hong et al., 2008; Fujioka et al., 2009; Pearce et al., 2010; Kopell et al., 2011). Our results strongly favor the idea that induced beta power associates with prediction rather than a simple response to rareness for two reasons. First, the induced power fluctuations of beta oscillation entrain to external isochronous tone sequences in the absence of deviants (Fujioka et al., 2012), which suggests that a primary function of induced beta power concerns temporal prediction rather than detecting rare events. Second, our analyses of standard tones showed that induced low-beta power responses were stronger after the onset of standard tones that were less likely to occur (i.e., the last standard tone occurring after an uninterrupted series of sequential standard tones, SpreD trials) than standard tones that were more likely to occur (i.e., standards occurring earlier in a sequence of standards, SbS trials). This confirms that increased induced low-beta power after tone onset reflects a process that is sensitive to the precision of prediction error.

Our results also suggest that the low-beta response is associated with precision-weighted prediction error while controlling possible effects of SSA. Previous studies on adaptation show that the neural response decreases to repeated tones, and that an increased response to the presentation of a new (rare) tone in an oddball context could reflect a release from this adaptation (e.g., Butler, 1968; Näätänen et al., 1988; Lanting et al., 2013). By selecting the deviant trials in the 10% and 20% sessions that had a matched number of standard tones preceding them we equated any effects of SSA between sessions. The results showed that the low-beta response to a deviant tone was larger in the 10% session than in the 20% session even after SSA was equated. Thus, the lower conditional likelihoods of encountering a deviant tone in the 10% than 20% session associate with a larger low-beta response on deviant trials. This analysis suggests that the low-beta response associates with precision-weighted prediction error although there may also have been a smaller effect of stimulus adaptation. Further research is needed on this question (e.g., see Herrmann et al., 2013, 2014, 2015).

A remaining question concerns the relation between prediction of rhythmic timing (Fujioka et al., 2012, 2015) and prediction precision for pitch, given that induced beta power is interactively modulated by both factors. Here we propose that timing and content (*when* and *what*) interact through attentional processing. Dynamic attending theory proposes that internal rhythmic entrainment to external temporal regularities is accomplished by a combination of self-sustained neural oscillation and the dynamic allocation of attention in the temporal dimension (Jones and Boltz, 1989; Large and Jones, 1999; Jones, 2010). The self-sustained oscillation acts as a time frame, and adapts its rate and phase to the external auditory rhythm. Attention increases at important time points such as the onset of beats, which is guided by the temporal prediction of the oscillatory time frame, and reflects temporal prediction for upcoming events during rhythmic entrainment. This attentional rhythmic entrainment is characterized as exogenous orienting (Jones et al., 2006; Nobre et al., 2007; Coull and Nobre, 2008; Jones, 2010), which is involuntary and automatic (Rohenkohl et al., 2011; Triviño et al., 2011; Correa et al., 2014). Further, an MEG study has shown that the mathematical model of dynamic attending theory predicts delta power activities generated in auditory cortex (Herrmann et al., 2016), suggesting that rhythmic attending modulates oscillatory activities in auditory cortex. In this way, it is possible that rhythmic beta power fluctuations representing attention to events with temporal regularity increase perceptual processing of the content of the input stream at predictable time points, such as beat onsets. The idea that beta oscillations reflect temporal attention is also consistent with converging evidence that similar processes occur in the motor system, where rhythmic temporal structure also plays a critical role (e.g., Nobre et al., 2007; Coull and Nobre, 2008; Morillon et al., 2015). This is particularly interesting given that an auditory rhythm sets up beta power oscillations not only in auditory cortex, but also in motor areas even though movement is not involved. Thus, beta power oscillations in response to a rhythmic auditory input have also been interpreted as reflecting communication between auditory and motor system in the cortex (Jenkinson and Brown, 2011; Fujioka et al., 2012, 2015; Kilavik et al., 2013).

A lack of concurrent behavioral measurements to confirm whether induced beta power modulates perceptual sensitivity is a limitation of the current study. Further experiments are needed to examine this directly. However, the evidence to date shows that increased beta power before a stimulus onset reflects enhanced predictive readiness and improves perceptual performance. Studies using an auditory spatial temporal order judgments task (Bernasconi et al., 2011), an auditory temporal delay detection task (Arnal et al., 2015), intensity detection task (Herrmann et al., 2016), pitch distortion detection task during music listening (Doelling and Poeppel, 2015), or an audiovisual temporal integration task (Geerligs and Akyürek, 2012), all show that when the beta band power happened to be larger in the pre-stimulus period, participants made more accurate judgments or had enhanced audiovisual integration compared to when beta power was smaller. Together, the results of these studies are consistent with our speculation that beta oscillations reflect attention (Wróbel, 2000; Buschman and Miller, 2007, 2009).

## Conclusion

We presented isochronous auditory oddball sequences containing occasional pitch deviants to show that induced beta power is sensitive to the content of the input during rhythmic entrainment. We replicated previous findings that induced beta power entrains to externally presented rhythms. More interestingly, we showed that unpredicted pitch deviants modulate beta power 200–300 ms after deviant tone onsets, and that the magnitude of the modulation reflects the deviant occurrence likelihood (precision-weighted prediction error). Our data show that induced beta power activities in auditory cortex are consistent with a role in sensory prediction for both *what* (pitch*)* will occur as well as *when* (rhythm) events will occur. The timing and nature of the beta power response to pitch deviants suggests that it reflects an attentional modulation. In conjunction with other research, we propose that predictions for *what* and *when* are dynamically processed through attentional networks, and that beta oscillations in auditory cortex reflect the functional significance of sensory prediction and prediction error processes.

## Author Contributions

AC, DB, and LT designed research; AC performed research; AC, DB, and LT contributed unpublished analytic tools; AC and DB analyzed data; AC, DB, and LT wrote the paper.

## Conflict of Interest Statement

The authors declare that the research was conducted in the absence of any commercial or financial relationships that could be construed as a potential conflict of interest.
